# Skeletal muscle depletion predicts survival of patients with advanced biliary tract cancer undergoing palliative chemotherapy

**DOI:** 10.18632/oncotarget.18345

**Published:** 2017-06-02

**Authors:** Kyoung-Min Cho, Hyunkyung Park, Do-Youn Oh, Tae-Yong Kim, Kyung Hun Lee, Sae-Won Han, Seock-Ah Im, Tae-You Kim, Yung-Jue Bang

**Affiliations:** ^1^ Department of Internal Medicine, Seoul National University College of Medicine, Seoul, Korea; ^2^ Cancer Research Institute, Seoul National University College of Medicine, Seoul, Korea; ^3^ Department of Internal Medicine, Graduate School, Kyung Hee University, Seoul, Republic of Korea

**Keywords:** biliary tract cancer, skeletal muscle depletion, weight change, BMI, prognosis

## Abstract

**Background:**

No prior study has investigated the dynamics of body weight with body muscle mass as a prognostic factor in advanced biliary tract cancer (BTC) patients undergoing palliative chemotherapy. We investigated whether low skeletal muscle mass affects survival in patients with BTC, with a co-analysis of body weight loss and body mass index (BMI).

**Results:**

By multivariate analysis, low skeletal muscle mass at diagnosis and decreased SMI during chemotherapy (*p* = 0.008 and *p* < 0.001, respectively) were poor prognostic factors for overall survival (OS). Subgroup analysis revealed that low skeletal muscle mass patients who were overweight or obese (BMI ≥ 25 kg/m^2^) showed worse OS (*p* < 0.001). Additionally, patients with both decreased BMI and SMI during chemotherapy had worse OS (*p* < 0.001). Furthermore, patients with decreased SMI had shorter survival regardless of change in BMI. However, for patients with SMI maintained during chemotherapy, decreased BMI had no effect on survival (*p* = 0.576).

**Materials and Methods:**

We consecutively enrolled 524 patients with advanced BTC who received palliative chemotherapy between 2003 and 2013. Total muscle cross-sectional area (cm^2^) at the L3 level assessed by computed tomography was analyzed. We defined low skeletal muscle mass as a skeletal muscle index (SMI) < 48.5 cm^2^/m^2^ (men) and < 39.5 cm^2^/m^2^ (women) using ROC curves.

**Conclusions:**

Low skeletal muscle mass, obesity and muscle depletion during palliative chemotherapy are meaningful prognostic factors in advanced BTC. Considering muscle depletion with weight change could help to more accurately predict prognoses of patients with BTC.

## INTRODUCTION

Biliary tract cancers (BTCs) are relatively rare tumors comprising intrahepatic cholangiocarcinoma (ICC), gallbladder cancer (GB Ca), extrahepatic biliary tract cancer (extrahepatic BTC) and ampulla of Vater cancer (AoV Ca) [1]. BTCs are associated with poor prognoses and significant cachexia [2].

Cachexia is a multi-organ syndrome associated anorexia, inflammation and increased muscle protein breakdown that cannot be fully reversed by nutritional support, and leads to progressive functional impairment [3, 4]. Cachexia is associated with reduced tolerance to anticancer therapy and reduced survival [5, 6]. Historically, cachexia has been defined by weight loss [7]. Weight loss during chemotherapy is associated with reduced response rates and increased toxicity [5]. However, body weight measurement may be underestimated in patients who have gained weight because of edema or tumor mass [3, 8]. Likewise, body mass index (BMI) is used clinically to define underweight and cachexia [9]. However, because obesity is now prevalent in industrialized nations, it may be unclear whether cancer patients with a BMI < 20 are cachectic [10]. Thus, cancer cachexia is a complex condition that is not yet fully defined [11].

Recently, low skeletal muscle mass (with or without loss of fat mass) – the concept of body composition –has been considered a main characteristic of cancer cachexia [12]. Several reports have shown low skeletal muscle mass as an independent poor prognostic factor for survival in patients with various cancers [13–15]. Furthermore, low skeletal muscle mass obesity is known to be a poor risk factor in several cancers in which loss of skeletal muscle and gain of adipose tissue can occur simultaneously [16, 17]. In addition, a recent report described that weight loss and muscle depletion share a poor prognosis [17]. Considering the above, recently, weight loss, body mass index (BMI) and low skeletal muscle mass the basis of the cancer cachexia definition.

In BTC, preoperative low skeletal muscle mass is closely related to mortality after resection [18, 19]. However, to date, reports on low skeletal muscle mass as a prognostic factor in advanced BTC are rare. Furthermore, to our knowledge, no study to date has investigated the dynamics of body weight and body muscle mass in advanced BTC patients undergoing palliative chemotherapy.

Hence, we investigated whether low skeletal muscle mass upon diagnosis and loss of skeletal muscle during palliative chemotherapy were associated with survival in patients with BTC. We also co-analyzed the dynamics of body weight and muscle depletion.

## RESULTS

### Patient characteristics

Of 598 initially-identified consecutive advanced BTC patients, this study included 524 patients with adequate CT images or existing BMI data undergoing palliative chemotherapy. The clinical characteristics of enrolled patients at initial assessment are shown according to sex in Table 1. The median age was 61 years (range, 26–87 years). There were 231 patients with ICC (44.1%), 162 patients with GB Ca (30.9%), 72 with extrahepatic BTC (13.7%), and 59 with AoV Ca (11.3%). Thirty-nine and nine tenths of a percent of patients were treated with gemcitabine/platinum, 37.6 % with 5-FU/platinum chemotherapy. The chemotherapies used are described in Supplementary Table 1.

**Table 1:** Clinical characteristics of patients

Characteristics		Men (*N* = 344)		Women (*N* = 180)		Total (*N* = 524)		*p* value men vs women
		*N*	%	*N*	%	*N*	%	
Age (years)	Mean	61		61		61		0.920^b^
	SD	9.5		9.2		9.4		
Primary tumor site								< 0.001^a^
	ICC	178	51.7%	53	29.4%	231	44.1 %	
	GB Ca	82	23.8%	80	44.4%	162	30.9 %	
	Extrahepatic BTC	49	14.2%	23	12.8%	72	13.7 %	
	AoV Ca	35	10.2%	24	13.3%	59	11.3 %	
ECOG PS	0–1	264	90.7%	140	84.8%	404	88.6 %	0.066^a^
	> 2	27	9.3%	25	15.2%	52	11.4 %	
SMI at diagnosis, Cm^2^/m^2^	Mean	48.58		41.24		46.02		< 0.001 ^b^
	SD	7.74		7.39		8.38		
Low skeletal muscle mass	Yes	150	51.0 %	62	39.5%	212	47.0 %	0.023 ^a^
	No	144	49.0 %	95	60.5%	239	53.0 %	
BMI at diagnosis, kg/m2	Mean	22.4		23.1		22.73		0.320 ^c^
	SD	2.94		2.83		2.91		
BMI at diagnosis	< 20 kg/m2	64	18.9 %	18	10.4 %	82	16.1 %	0.042 ^a^
	20–24.9 kg/m2	202	59.8 %	110	64.0 %	312	61.2 %	
	> 25 kg/m2	72	21.3 %	44	25.6 %	116	22.7 %	
Disease status	Locally advanced	98	28.5 %	66	36.7 %	164	31.3 %	0.060
	Metastatic	246	71.5 %	114	63.3 %	360	68.7 %	
Sites of metastases	Lymph Node	182	52.9 %	113	62.8 %	295	56.3%	0.033
	Liver	161	46.8 %	89	49.4 %	250	47.7 %	0.582
	Peritoneum	114	33.1 %	52	28.9 %	166	31.7 %	0.374
	Lung	39	11.3 %	19	10.6 %	58	11.1 %	0.884
	Bone	17	4.9 %	7	3.9 %	24	4.6 %	0.665

ICC,intrahepatic cholangiocarcinoma;GB Ca,gallbladder cancer;extrahepatic BTC, extrahepatic biliary tract cancer;AoV Ca,ampulla of Vater cancer; ECOG PS, Eastern Cooperative Oncology Group performance status; BMI, body mass index; SMI, skeletal muscle index

† Low skeletal muscle mass: males < 48.5 cm^2^/m^2^, females < 39.5 cm^2^/m^2^.

^*a*^*p* values were calculated using the Fisher exact test.

^*b*^*p* values were calculated using the Mann-Whitney *U* test.

^*c*^*p* values were calculated using the independent *t*-test.

**Figure 1:**
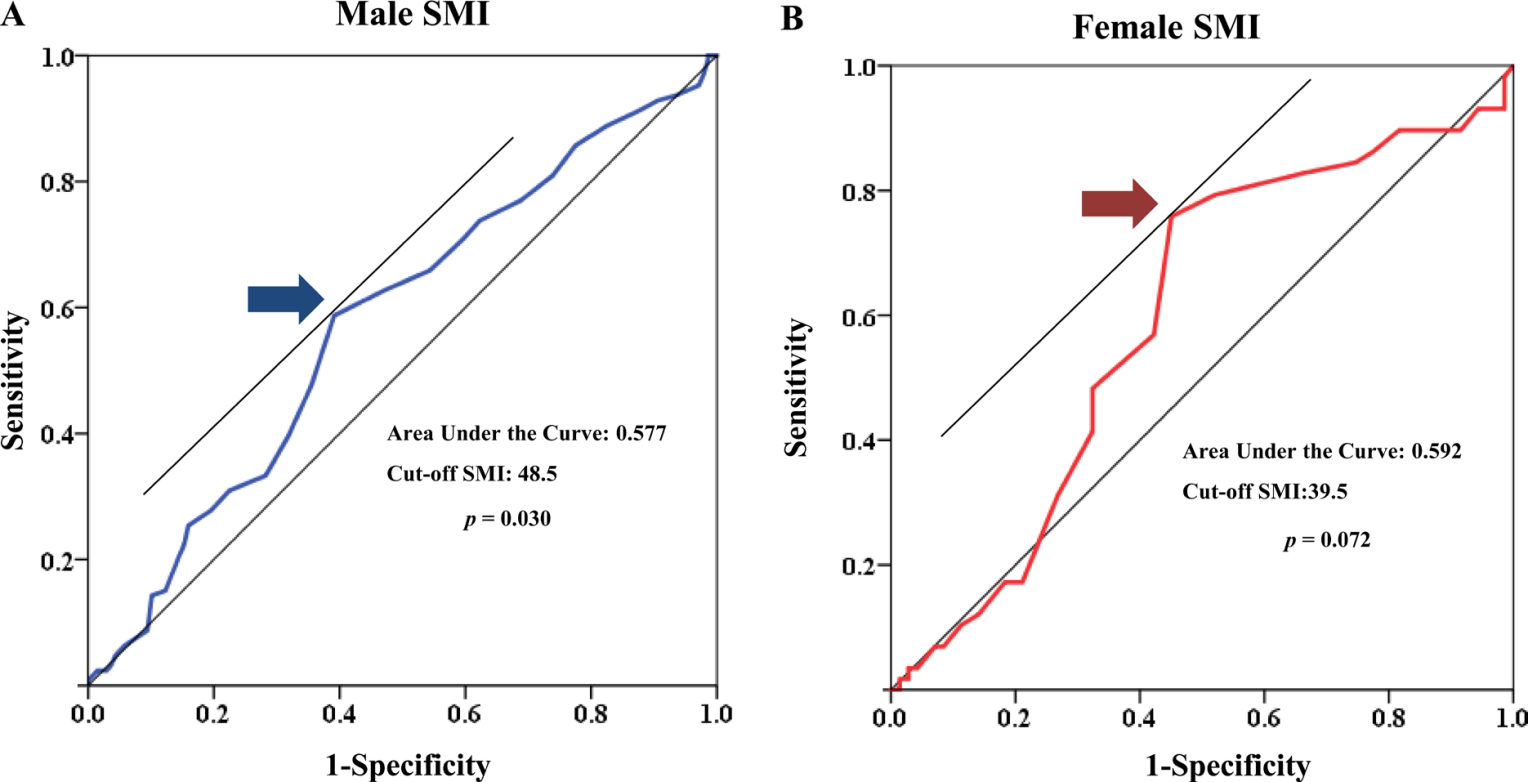
Receiver operating characteristic (ROC) curve of low muscle mass for both sexes

The cutoff values for low skeletal muscle mass as determined via ROC curves were < 48.5 cm^2^/m^2^ for men and < 39.5 cm^2^/m^2^ for women (Figure 1).

### Body composition

There were no significant differences in mean BMI values or the distribution of patients in BMI subgroups based on sex. The proportion of overweight or obese (BMI ≥ 25 kg/m^2^) men and women was 21.3% and 25.6%, respectively.

**Figure 2:**
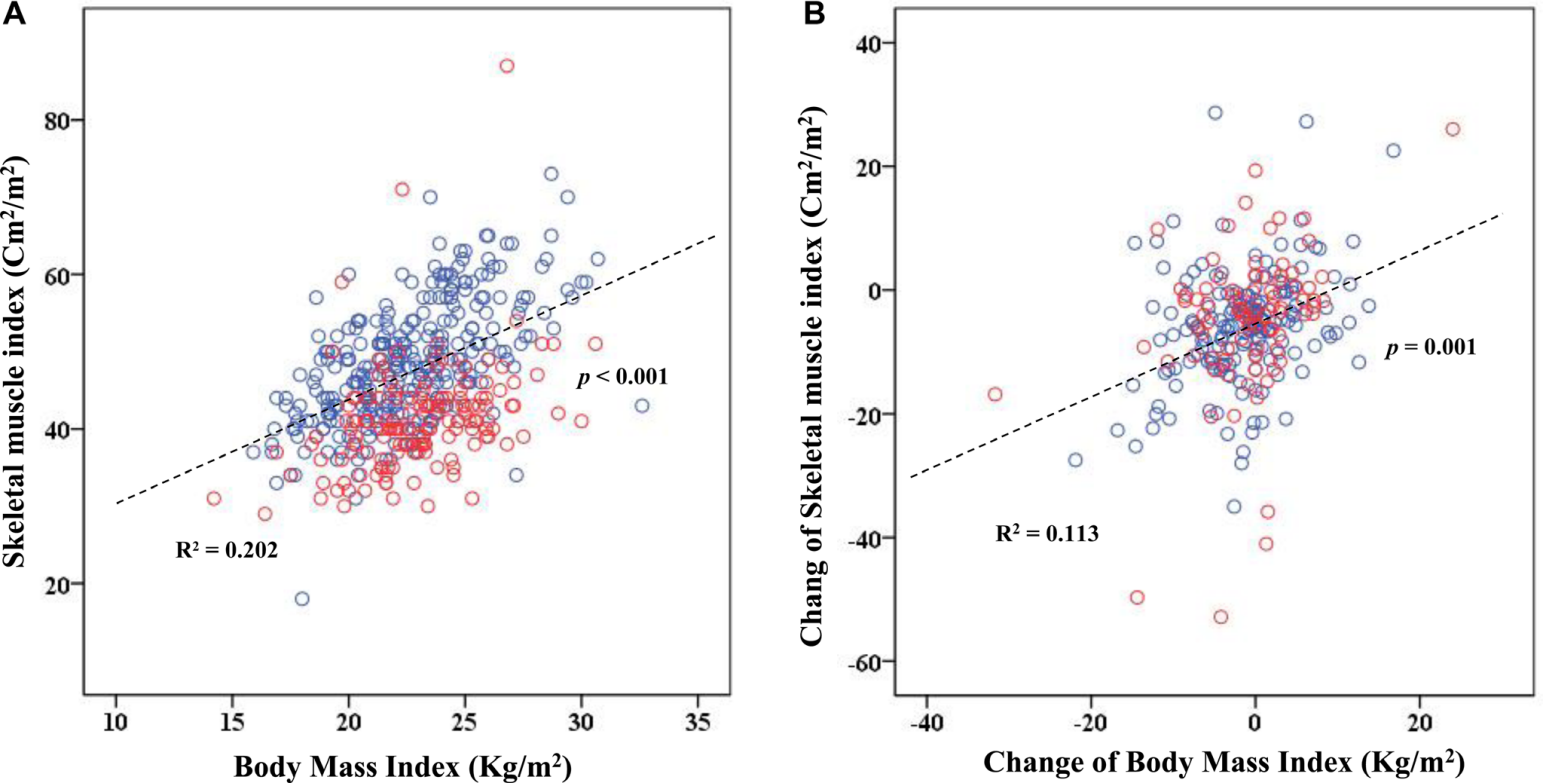
Scatter plot for skeletal muscle index (SMI) and body mass index (BMI) (**A**) Relationship between SMI and BMI. (Pearson *r* = 0.449, *p* < 0.001 for all patients; men, *r* = 0.593, *p* < 0.001; women, *r* = 0.381, *p* < 0 .001). (**B**) Relationship between changes in SMI and BMI. (Pearson *r* = 0.336, *p* < 0.001 for all patients; men, *r* = 0.328, *p* < 0.001; women, *r* = 0.349, *p* = 0.001).

The mean SMI values (± SD) for men and women were 48.58 ± 7.74 cm^2^/m^2^ and 41.24 ± 7.39 cm^2^/m^2^, respectively (*p* < 0.001). Additionally, there were significant differences in the distributions of men and women in SMI subgroups (*p* = 0.023). The proportion of patients with low skeletal muscle mass upon diagnosis was 51.0% and 39.5% for men and women, respectively (Table 1). The distribution of low skeletal muscle mass was broader in men. There were 16 (3.1%) patients with low skeletal muscle mass and obesity (overweight or obese patients with low skeletal muscle mass) and 55 (10.5%) with obvious cachexia (BMI < 20 kg/m^2^ with low skeletal muscle mass; Supplementary Table 2). SMI upon diagnosis was significantly correlated with BMI upon diagnosis in both sexes (Pearson *r* = 0.449, *p* < 0.001 for all the patients; males, *r* = 0.593, *p* < 0.001; females, *r* = 0.381, *p* < 0 .001; Figure 2).

During palliative chemotherapy, mean BMI and SMI losses were 1.63 Kg/m^2^ and 5.35 cm^2^/m^2^, respectively (Table 2). Mean BMI loss was not significantly different between men and women (*p* = 0.109). However, mean SMI loss was significantly greater in men than in women (5.72 cm^2^/m^2^ vs 4.61 cm^2^/m^2^; *p* = 0.031).

**Table 2:** Changes in body composition during palliative chemotherapy

Characteristics	Men		Women		Total		*p* value
	*N*	%	*N*	%	*N*	%	
Change in BMI Mean	−1.95		−1.021		−1.63		0.109^b^
Kg/m^2^ (%) SD	6.36		7.01		6.59		
Change in BMI							0.518^a^
Decreased	35	17.7%	15	14.4%			
Maintained	163	82.3%	89	85.6%			
Change in SMI Mean	−5.72		−4.61		−5.35		0.031^b^
Cm^2^/m^2^ (%) SD	9.52		12.02		10.40		
Change in SMI							0.197^a^
Decreased	75	37.5%	29	29.6%			
Maintained	125	62.5%	69	70.4%			

BMI, body mass index; SMI, skeletal muscle index; SD, standard deviation.

^†^Change in BMI: maintained ≥ −7% kg/m^2^, decreased < −7% kg/m^2^

^‡^Change in SMI: maintained ≥ −7 % cm^2^/m^2^; decreased < −7 %cm^2^/m^2^.

^*a*^*p* values were calculated using the Fisher exact test.

^*b*^*p* values were calculated using the Mann-Whitney *U* test.

### Factors related to a decrease in skeletal muscle index during chemotherapy

Logistic regression analysis revealed that primary tumor origin, ECOG and decreased BMI during chemotherapy were significantly associated with decreased SMI during chemotherapy (Supplementary Tables 3 and 4).

### Treatment outcome and factors related to overall survival

Median OS and PFS of all patients were 9.00 months (95% CI, 8.31–9.69 months) and 4.30 months (95% CI, 3.88–4.72 months), respectively. The objective response and disease control rates were 19.5% and 68.6%, respectively.

**Figure 3:**
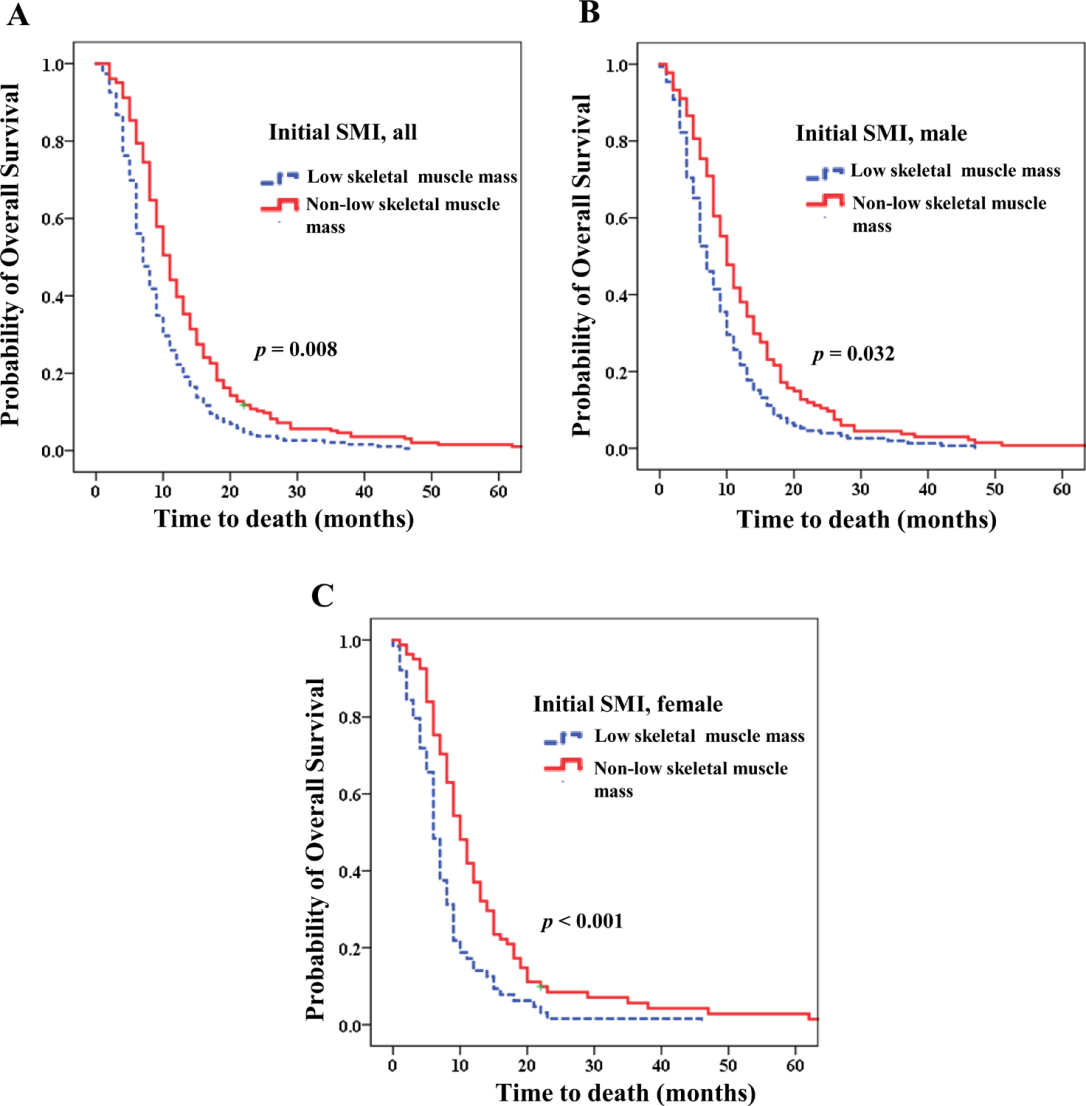
Overall survival according to skeletal muscle index (SMI) Initial low skeletal muscle mass was related to poorer prognosis (**A**) for the entire patient population (Hazard ratio (HR) 1.569; *p =* 0.008); (**B**) for men (HR 1.511; *p* = 0.032); (**C**) for women (HR 4.042; *p <* 0.001).

**Figure 4:**
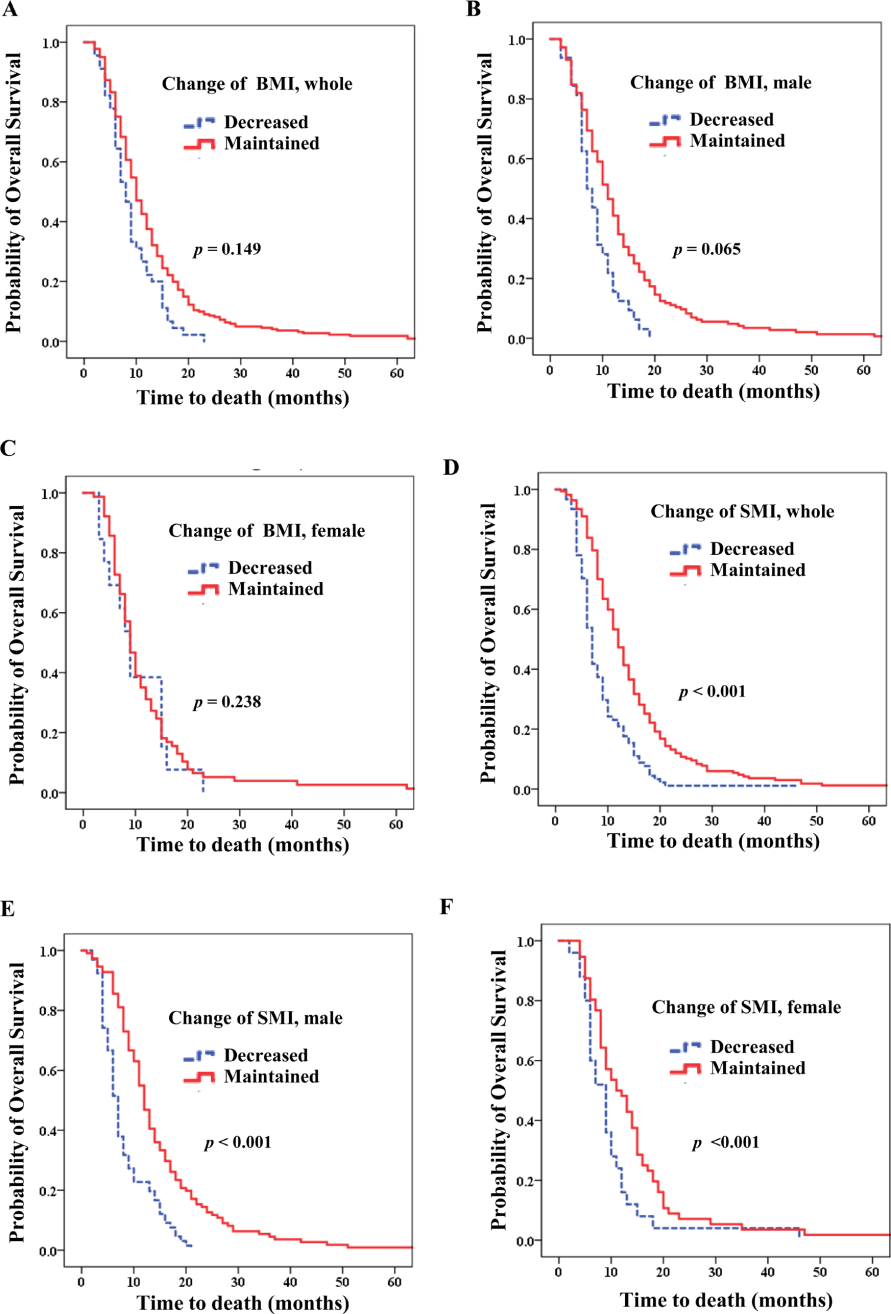
Overall survival according to change in body mass index (BMI) and skeletal muscle index (SMI) Decrease in BMI (**A**) for the entire patient population (Hazard ratio (HR) 1.402; *p* = 0.116); (**B**) for men (HR 1.584; *p* = 0.065); (**C**) for women (HR 0.571; *p* = 0.238). Decrease in SMI (**D**) for the entire patient population (HR 2.580*; p* < 0.001); (**E**) for men (HR 2.370; *p* < 0.001); (**F**) for women (HR 4.042; *p* < 0.001).

Table 3 shows the effects of multiple clinical factors on OS. Via multivariate analysis, low skeletal muscle mass (HR, 1.569; 95% CI, 1.127–2.186; *p* = 0.008; Figure 3A) and decreased SMI (HR, 2.580; 95% CI, 1.860–3.579; *p* < 0.001; Figure 4D) were associated with poor survival. However, BMI upon initial diagnosis and decreased BMI were not related to OS (Figure 4A).

**Table 3:** Factors associated with OS

		Univariate analysis			Mutivariate		
		OS , months	95% CI	*p* value	HR	95% CI	*p* value
Gender	Male	9.00	8.110–9.890	0.837	1		0.793
	Female	9.00	8.039–9.961		0.955	0.679–1.345	
Age	< 60	10.00	9.123–10.877	0.033	1		0.192
	> 60	8.00	7.142–8.858		1.223	0.904–1.654	
Primary tumor site				0.106			0.086
	ICC	9.00	7.993–10.007		1		
	GB Ca	9.00	7.924–10.076		0.937	0.659–1.334	0.720
	Extrahepatic BTC	9.00	7.829–10.171		0.683	0.440–1.060	0.089
	AoV Ca	12.00	8.863–15.137		0.559	0.328–0.951	0.032
ECOG PS	0–1	10.00	9.186–10.814	< 0.001	1		0.445
	> 2	5.00	3.647–6.353		1.261	0.695 –2.287	
1 st chemotherapy	Gemcitabine- platinum	8.67	7.422–9.911	0.085	1		0.399
	FU- platinum	9.40	8.371–10.429		0.826	0.604 –1.131	0.234
	Others	8.53	7.591–9.476		1.081	0.657 –1.778	0.759
Extended status	Locally advanced	8.73	7.94–9.523	0.945	1		0.991
	Metastasis	8.90			0.998	0.716–1.391	
Low skeletal muscle mass	Yes	7.00	6.003–7.997	< 0.001	1.569	1.127–2.186	0.008
	No	11.00	10.007–11.993		1		
BMI at diagnosis				0.345			0.031
	< 20 kg/m2	8.00	6.338–9.662		1		
	20–24.9 kg/m2	9.00	8.067–9.933		0.701	0.453–1.084	0.110
	> 25 kg/m2	9.00	7.879–10.121		1.162	0.679–1.989	0.585
Change in BMI	Decreased	8.00	6.594–9.406	0.003	1.402	0.920–2.138	0.116
	Maintained	10.00	8.909–11.091		1		
Change in SMI	Decreased	7.00	6.291–7.709	< 0.001	2.580	1.860–3.579	< 0.001
	Maintained	12.00	10.776–13.224		1		
Best response	Controlled	11.00	10.007–11.993	< 0.001	1		< 0.001
	Progression	6.00	5.335–6.665		2.647	1.908–3.672	

ICC, intrahepatic cholangiocarcinoma;GB Ca, gallbladder cancer;extrahepatic BTC, extrahepatic biliary tract cancer;AoV Ca,ampulla of Vater cancer; ECOG PS, Eastern Cooperative Oncology Group performance status; BMI, body mass index; SMI, skeletal muscle index; OS, overall survival; HR, hazard ratio; CI, confidential interval. ^†^ controlled: complete response, partial response, and stable disease.

For men, low skeletal muscle mass (median OS of 7.0 vs 10.0 months; HR 1.511; 95% CI, 1.037–2.200; *p* = 0.032; Figure 3B) and decreased SMI (median OS of 7.0 vs 12.0 months; HR, 2.370, 95% CI, 1.603–3.504; *p* < 0.001; Figure 4E) were strongly related to poorer prognosis. Additionally, patients who were overweight upon diagnosis (median OS 8.0 vs 9.0 months; *p* = 0.029) showed poorer prognoses than those who were normal weight. However, decreased BMI did not influence OS (*p* = 0.065; Figure 4B).

For women, both low skeletal muscle (median OS 6.00 vs 11.00 months; HR,4.042; 95% CI, 2.007–8.140; *p* < 0.001; Figure 3C) and decreased SMI (median OS 9.00 vs 11.00 months; HR, 4.042; 95% CI, 2.007–8.140; *p* < 0.001; Figure 4F) were significantly associated with poorer prognosis. However, BMI upon diagnosis and decreased BMI were not related to OS (*p* = 0.207 and 0.238, respectively; Table 4; Figure 4C).

**Table 4:** Body composition analyses for predictors of overall survival by gender groups

		Male				Female			
		OS, months	HR	95% CI	*p*^a^value	OS, months	HR	95% CI	*p*^a^ value
Low skeletal muscle mass	Yes	7.00	1.511	1.037–2.200	0.032	6.00	4.042	2.007–8.140	< 0.001
	No	10.00	1			11.00	1		
Initial BMI at diagnosis					0.029				0.207
	< 20 kg/m2	8.00	1			7.00	1		
	20–24.9 kg/m2	9.00	0.600	0.365–0.985	0.043	9.00	0.730	0.230–2.315	
	>25 kg/m2	8.00	0.995	0.531–1.864	0.988	9.00	1.577	0.454–5.480	
Change in BMI	Decreased	7.00	1.584	0.971–2.584	0.065	9.00	0.571	0.226–1.447	0.238
	Maintained	11.00	1			9.00	1		
Change in SMI	Decreased	7.00	2.370	1.603–3.504	< 0.001	9.00	4.042	2.007–8.140	< 0.001
	Maintained	12.00	1			11.00	1		
Disease status	Locally advanced	9.00	1		0.925		1		0.612
	Metastatic	8.90	1.020	0.670–1.554			0.854	0.465–1.570	

BMI, body mass index; SMI, skeletal muscle index; OS, overall survival; HR,harzard ratio; CI, confidential interval.

^*a*^*p* values were calculated using the Cox-proportional hazard model, adjusted with age, Primary tumor site, and PS.

Low skeletal muscle mass and decrease in SMI were significantly associated with poorer OS than non-lower skeletal muscle mass and maintained in SMI via subgroup analysis according to ICC (*p* < 0.001 and 0.002, respectively; Supplementary Table 5). The number of patients with both initial SMI and BMI as well as progression SMI and BMI was 248. When we performed an analysis of these patients, the results were similar (Supplementary Table 6).

### Correlation between BMI and SMI upon diagnosis

**Figure 5:**
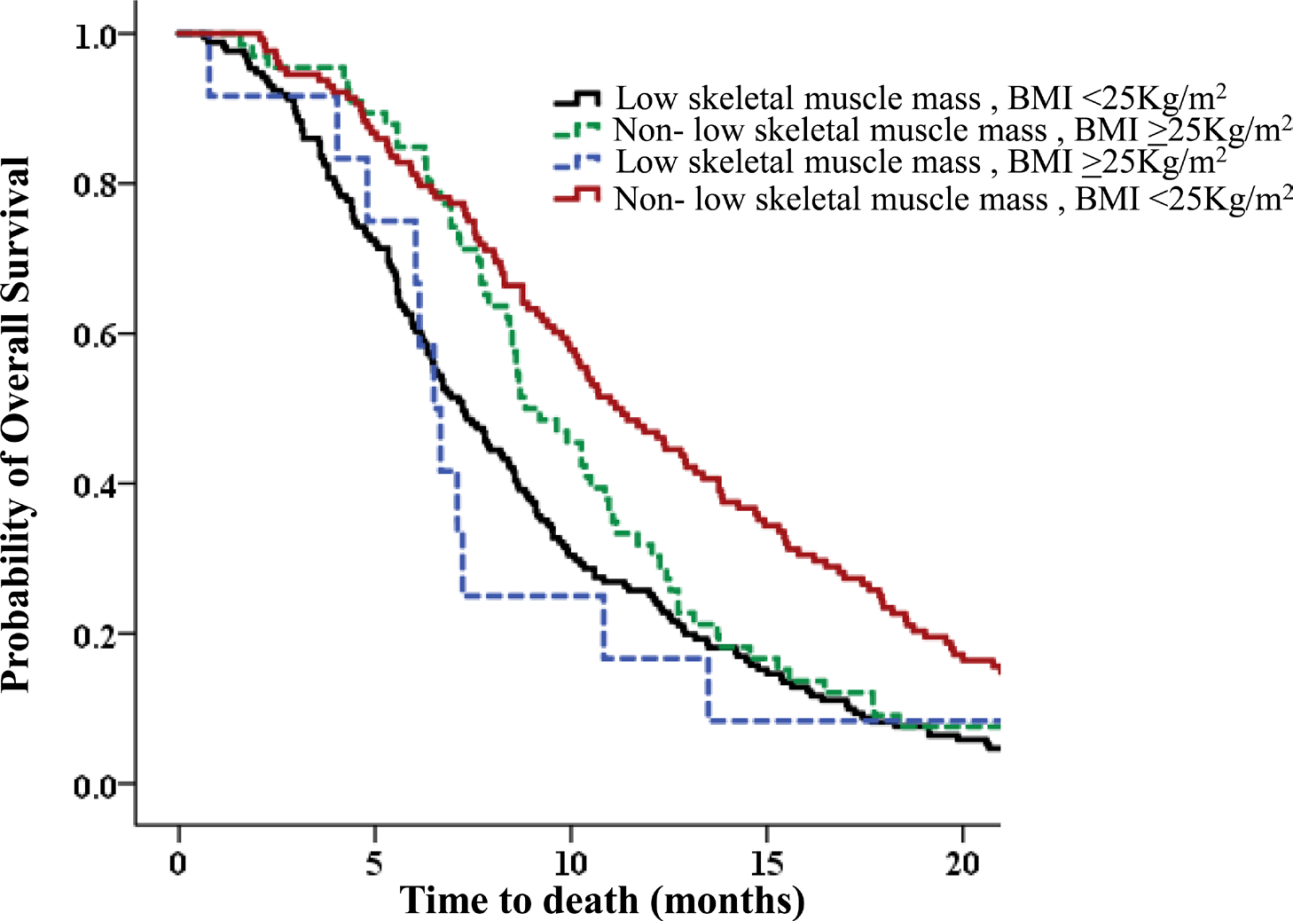
Overall survival (OS) according to correlation between body mass index (BMI) and skeletal muscle index (SMI) upon diagnosis Patients with low skeletal muscle mass who were overweight or obese had the poorest OS. Patients with low skeletal muscle mass who were overweight or obese had a 4.0-month shorter average OS than non-low skeletal muscle mass patients who were not overweight or obese (median OS 11.0 months vs 6.0 months; HR,2.130; *p* < 0.001).

Further analysis categorizing BMI upon diagnosis into overweight or obese (≥ 25 kg/m^2^) or not (< 25 kg/m^2^) and SMI upon diagnosis into non-low skeletal muscle mass or low skeletal muscle mass was performed. The results revealed that low skeletal muscle mass patients who were overweight or obese had the poorest OS (Table 5). Compared to a median OS of 11.0 months for patients with non-low skeletal muscle mass who were not obese, low skeletal muscle was a poor prognostic indicator independent of BMI. Low skeletal muscle mass patients had a 4.0-month shorter OS than non-low skeletal muscle mass patients who were not overweight or obese (median OS 11.0 vs 7.0 months; HR, 1.854; *p* < 0.001) (Figure 5). For patients with low skeletal muscle mass, being overweight or obese had no effect on survival (*p* = 0.803). For patients with non-low skeletal muscle mass, being overweight or obese worsened survival (*p* = 0.002). Similar results were observed for men. For men with non-low skeletal muscle mass, being overweight or obese had a negative effect on survival, although this result did not achieve statistical significance.

**Table 5:** Correlation of BMI and SMI at diagnosis

		BMI at diagnosis			
		< 25 kg/m^2^ (*N* = 394)	> 25 kg/m^2^ (*N* = 116)		
SMI at diagnosis	Low skeletal muscle mass, *N* = 212	HR:1.854 (95% CI:1.430–2.403) *p* < 0.001^a^, OS = 7.00 months, *N* = 190	HR:2.130 (95% CI:1.135–3.997) *p* = 0.019^a^, OS = 6.00 months, *N* = 16	*p* = 0.803^a,c^	*p* < 0.001^a^
	Non-low skeletal muscle mass, *N* = 239	HR:1 reference OS=11.00 months, N=150	HR:1.630 (95% CI:1.178–2.256) *p* = 0.003^a^, OS = 9.00 months, *N* = 81	*p* = 0.002^a,d^	
SMI males, *N* = 344	Low skeletal muscle mass, *N* = 150	HR:1.723 (95% CI:1.257–2.362 ) *p* = 0.001^b^, OS= 8.00 months, N=138	HR: 2.598 (95% CI:1.217–5.548) *p* = 0.014^b^, OS = 6.00 months, *N* = 10	*p* = 0.314^b,c^	*p* = 0.003^b^
	Non-low skeletal muscle mass, *N* = 144	HR:1 Reference OS= 11.00 months,N=90	HR:1.532 (95% CI:1.006–2.333) *p* = 0.047 ^b^, OS = 10.00 months, *N* = 50	*p* = 0.07^b,d^	
SMI female, *N* = 180	Low skeletal muscle mass, *N* = 62	HR: 2.526 (95% CI:1.518–4.204) *p* < 0.001^b^, OS=6.00 months, N=52	HR:1.792 (95% CI:0.517–6.204) *p* = 0.357 ^b^, OS = 7.00 months, *N* = 6	*p* = 0.840^b,c^	*p* = 0.005^b^
	Non-low skeletal muscle mass, *N* = 95	HR: 1 Reference OS=12.00 months, N=60	HR: 1.919 (95% CI:1.091–3.376) *p* = 0.024 ^b^,OS = 9.00 months, *N* = 31	*p* = 0.032 ^b,d^	

^*a*^*p* values were calculated using the Cox-proportional hazard model, Primary tumor site , age and PS,gender.

^*b*^*p* values were calculated using the Cox-proportional hazard model, adjusted with age, Primary tumor site and PS.

^*c*^*p* values were calculated within sarcopenia group. ^d^*p* values were calculated within nonsarcopenia group.

Women who had low skeletal muscle mass and were not overweight demonstrated the poorest OS. However, little difference was observed in OS between low skeletal muscle mass patients who were not vs were overweight (6.0 months vs 7.0 months). When we performed similar analysis focusing on ICC, the results were similar (Supplementary Table 7).

### Correlation of changes BMI and SMI during chemotherapy

Decreased BMI was an important factor associated with a decrease in SMI (HR, 2.650; *p* = 0.022; Supplementary Table 3). Additionally, the distribution of change in BMI was strongly correlated with the distribution of change in SMI in both sexes (Pearson *r* = 0.336, *p* < 0.001 for all the patients; males, *r* = 0.328, *p* < 0.001; females, *r* = 0.349, *p* = 0.001; Figure 2).

Analysis of patients in SMI and BMI subgroups (SMI and BMI both maintained during chemotherapy, SMI maintained and BMI decreased, SMI decreased and BMI maintained, and SMI and BMI both decreased) revealed that patients with both decreased BMI and SMI had the shortest survival (*p* < 0.001; Table 6). Furthermore, patients with decreased SMI had shorter survival regardless of change in BMI (*p* < 0.001; Table 6).

**Table 6:** Correlation of changes in BMI and SMI

		BMI			
		Decreased (*N* = 50)	Maintained (*N* = 252)		
SMI	Decreased (*N* = 104)	HR:2.779 (CI 95% 1.587–4.866) *p* < 0.001^a^, OS = 7.00 months, *N* = 20	HR:2.186 (CI 95% 1.555–3.074) ;*p* < 0.001^a^, OS = 7.00 months, *N* = 69	*p* = 0.430^a,c^	*p* < 0.001^a^
	Maintained (*N* = 194)	HR:1.176 (CI 95% 0.666–2.075) ;*p* = 0.576^a^ , OS = 11.00 months, *N* = 18	HR:1; Reference OS = 12.00 months, *N* =139	*p* = 0.714^a,d^	
Males (*N* = 344)	Decreased (*N* = 75)	HR:3.380 (CI 95% 1.729–6.609) ;*p* < 0.001^b^, OS = 6.00 months, *N* = 15	HR:2.333(CI 95% 1.523–3.575) ;*p* < 0.001^b^, OS = 6.00 months, *N* = 48	*p* = 0.459^b,c^	*p* < 0.001^b^
	Maintained (*N* = 125)	HR:1.626 (CI 95% 0.815–3.243); *p* = 0.167 ^b^, OS = 11.00 months, *N* = 13	HR:1; Reference OS = 12.00 months, *N* = 88	*p* = 0.241 ^b,d^	
Female (*N* = 180)	Decreased (*N* = 29)	HR:2.079 (CI 95% 0.513–8.426) *p* = 0.305 ^b^, OS = 7.00 months, *N* = 5	HR:2.182 (CI 95% 1.049–4.542) *p* = 0.037 ^b^, OS = 7.00 months, *N* = 21	*p* = 0.109 ^b,c^	*p* = 0.067^b^
	Maintained (*N* = 69)	HR: 0.454 (CI 95% 0.145–1.419) *p* = 0.174 ^b^, OS = 15.00 months, *N* = 5	HR:1 Reference OS = 10.00 months, *N* = 51	*p* = 0.278 ^b,d^	

^*a*^*p* values were calculated using the Cox-proportional hazard model, adjusted with gender, age, primary tumor site, PS, BMI at diagnosis, and sarcopenia.

^*b*^*p* values were calculated using the Cox-proportional hazard model, adjusted with age, PS, primary tumor site BMI at diagnosis, and sarcopenia.

^*c*^*p* values were calculated within decreased SMI group. ^d^*P* values were calculated within maintained SMI group.

Compared with a median OS of 12.0 months for patients with both SMI and BMI maintained during chemotherapy, decreased SMI was a poor prognostic indicator when BMI was maintained (median OS 7.0 months; HR, 2.186; *p* < 0.001; Table 6). However, for patients with SMI maintained during chemotherapy, decreased BMI had no effect on survival (*p =* 0.576). Analyses according to each sex (men, women) showed similar results.

## DISCUSSION

In this study, we found that low skeletal muscle mass upon diagnosis and skeletal muscle depletion during chemotherapy predicted worse survival for patients with advanced BTC.

The influence of BMI upon diagnosis and change of weight (BMI) during chemotherapy is not well understood. One report has shown that obesity is associated with a high risk of BTC [20]. However, no report has analyzed BMI in BTC as predicting OS prognosis. Our study revealed that initial BMI upon diagnosis is not significantly associated with OS. However, based on sex analysis, a BMI upon diagnosis > 25 kg/m^2^ confers a poor prognosis for men. Furthermore, within the non-low skeletal muscle mass patient group, being overweight or obese (BMI > 25 kg/m^2^) leads to a worse prognosis (Table 5). Considering the above, although initial BMI is not prognostic in BTC, when considered along with the low skeletal muscle mass state, BMI might help to predict prognosis in BTC.

Our study showed that low skeletal muscle mass and decreased SMI during chemotherapy were associated with poor survival. Subdivision analysis of men & women showed similar results. Our study revealed that low skeletal muscle mass and obesity confers the poorest prognosis (HR, 2.130; *p* < 0.001; Table 5). This result is in line with that of a previous report, which showed that low skeletal muscle mass and obesity confers a poor prognosis [17]. Our study revealed that women with low skeletal muscle mass and obesity did not have the poorest survival. However, the OS for patients with low skeletal muscle mass and obesity in women, was similar to that of the women demonstrating the poorest OS (7.0 vs 6.0 months; Table 5). Additionally, in our study, very few women were enrolled who had low skeletal muscle mass and were obese (*N* = 6), mainly because the prevalence of obesity is lower in Asian than in Western populations.

We showed that the decreased SMI during chemotherapy with decreased BMI was associated with a worse survival outcome (Table 6). Furthermore, while the decrease in BMI during chemotherapy did not significantly affect survival in the patients with maintained SMI (*p* = 0.714), a decrease in SMI was associated with reduced survival in the patients with maintained BMI (HR, 2.186; *p* < 0.001; Table 6). Analysis by sex showed similar results. Additionally, decreased BMI did not confer a significantly worse prognosis via multivariate analysis. Considering above, muscle depletion would be a more accurate prognostic factor than weight loss. However, considering muscle depletion along with weight loss might help to more accurately predict heterogeneous prognoses in BTC.

Our study also showed that muscle depletion varies according to the tumor origin of BTC (*p* = 0.008; Supplementary Table 3). The AoV Ca shows the lowest SMI decrease as compared to other primary tumors; thus, differences in OS between primary tumors in BTC might be associated with muscle depletion to some extent.

One previous report shows that median OS was significantly shorter in patients with low skeletal muscle mass (7.0 vs 2.0 months) in BTC [21]. However, this study analyzed only 29 patients and employed univariate survival analysis [21]. In contrast, our study enrolled a larger number of patients and employed multivariate survival analysis. Additionally, co-analysis was performed in our study for BMI, weight change and muscle change during chemotherapy.

Low skeletal muscle mass and obesity are common in advanced cancer and independently predict immobility and mortality [16]. Decreased quality of life, decreased disease-free survival, increased risk of cerebral vascular disease, reduced bone mass, and increased risk of fractures at multiple sites are likely related to both sarcopenia and obesity [22, 23]. Furthermore, patients with low skeletal muscle mass or low skeletal muscle mass obesity seem prone to toxic effects during chemotherapy [24–26], requiring dose reductions or treatment delays [26]. Most chemotherapy drugs are administered based on body surface area. Such practice ignores the large individual variability in muscle mass such that patients with lean body mass may receive a large concentration of drug, increasing drug toxicity [16].

Pathophysiology is characterized by a negative protein and energy balance driven by a variable combination of reduced food intake and abnormal metabolism [3]. IL-6 and TNF-α play important roles in inducing low skeletal muscle mass [27], mainly through stimulating muscle proteolysis and myocyte apoptosis [28]. Furthermore, deteriorated mitochondria are known to be associated with a loss of skeletal muscle integrity [29].

No treatment that leads to a gain of muscle mass in cancer patients has been defined to date. However, one recent report has shown that selumetinib promotes muscle gain in BTC [30]. Additionally, although there is little evidence of an effect of physical exercise on muscle strength, there is one report that showed ameliorated muscle proteolysis in experimental cancer cachexia [31]. Thus, increasing physical activity and muscle loading would be helpful for cancer patients.

The main limitations of our study were its retrospective design and the threshold values used to identify low skeletal muscle mass. The most frequently used cutoffs of sarcopenia in the Western population are 7.26 kg/m^2^ for men and 5.45 kg/m^2^ for women by dual-energy x-ray absorptiometry (DEXA) [32, 33]. Recently, these values were converted to CT measurements, which are correlated with the L3 skeletal muscle area and lumbar SMI (cm^2^/m^2^) [34–36]. However, low skeletal muscle mass defined by the cutoffs from previous Western studies may not be appropriate for a diagnosis of sarcopenia in the Asian population [37]. Therefore, we used ROC curve cut offs, the same method that was used in a previous report in Korea [13]. However, cutoff values in our study are different from those of previous reports on pancreatic cancer [13]. This observed difference in cutoff value may be attributable to different cancers, obese patients, or sex composition of the analyzed patient group. Thus, to validate our study, we used the cutoff value used in a previous study (52.4 cm^2^/m^2^ for men and 38.5 cm^2^/m^2^ for women) [38]; the results were similar to those of our original analysis (Supplementary Table 8). In addition, when we validated the current results using an ongoing study (selection of patients from “Biomarker discovery for predicting anti-cancer treatment response and prognosis in advanced gastric cancer, pancreatic cancer, biliary tract cancer and neuroendocrine tumor through prospective collection (from 2013) of human material: IRB No. H1306-069-497), we found that patients with low skeletal muscle mass had shorter OS than patients with non-low skeletal muscle mass; however, this result was not significant (Supplementary Table 9). Considering that is and ongoing prospective study, only sixty-two patients was analyzed; thus, further study and more patients are needed for a more robust analysis.

Finally, our study produced somewhat different results as compared to studies including other tumors, such as lung cancer [39]. However, ours represents a well-structured study on BTC, and provides a basis for further study.

In conclusion, low skeletal muscle, low skeletal muscle mass obesity and muscle depletion during palliative chemotherapy are meaningful prognostic factors in advanced BTC. Although initial BMI upon diagnosis might not be a prognostic factor, considering BMI along with low skeletal muscle mass state might aid in predicting prognosis. In addition, considering muscle depletion with weight change could be help to predict a more accurate prognosis in BTC. Thus, assessment of body composition should be considered when treating patients with advanced BTC.

## MATERIALS AND METHODS

### Patients

We retrospectively enrolled consecutive patients with advanced biliary tract cancer (BTC) who were treated at Seoul National University Hospital (Seoul, South Korea) between 2003 and 2013. All patients fulfilled the following criteria: (1) BTC was histologically confirmed; (2) BTC was unresectable and recurrent; (3) body weight and height were recorded at the time of unresectable/recurrent BTC diagnosis; and (4) an abdominal computed tomography (CT) scan was performed within the 30 days prior to starting the first cycle of palliative chemotherapy. The medical records of each patient were reviewed and the following data were extracted: BMI, sex, age, anatomical origin of tumor, tumor localization, pathology, progression-free survival (PFS) and overall survival (OS).

### Skeletal muscle mass measurement

CT scans performed upon diagnosis and progression date after first-line chemotherapy were used to quantify the initial area of L3 skeletal muscle. Two adjacent axial images within the same series, the third lumbar vertebra (L3), were selected for analysis of total muscle cross-sectional area (cm^2^) and averaged for each patient [17, 34, 40, 41]. Muscles were quantified within a Hounsfield unit (HU) range of -29 to 150 HU19 using Rapidia 3D software (v2.8; INFINITT Healthcare, Seoul, Korea) [13]. Muscle area was normalized to height in meters squared (m^2^) and reported as lumbar skeletal muscle index (SMI; cm^2^/m^2^) [13, 16, 17]. We used the area under the receiver operating characteristic (ROC) curve to determine the cutoff values for low skeletal muscle mass for both sexes.

### Statistical analyses

Fisher’s exact and independent *t*-tests were used for continuous variables to compare baseline characteristics between two groups of patients. Logistic regression was used to identify factors associated with a decrease in SMI during chemotherapy. OS was calculated from the first day of palliative chemotherapy to the day of death, and PFS was calculated from the first day of palliative chemotherapy to the first day of progression or last follow-up. OS and PFS were estimated using the Kaplan-Meier method. A log-rank test was used to assess differences in OS and PFS across groups. The effect of patient characteristics and other prognostic factors, such as SMI or a decrease in SMI, on survival was evaluated using Cox proportional hazard models (univariate and multivariate analyses) and the resultant hazard ratio (HR) and 95% confidence intervals (95% CIs). A *p* value of 0.05 or less was considered statistically significant. All analyses were performed using SPSS software for Windows (version 20; IBM SPSS, Somers, NY, USA).

### Ethics

The study protocol was reviewed and approved by the Institutional Review Board of Seoul National University Hospital (IRB No.H-1306-109-500). The study was conducted according to guidelines (Declaration of Helsinki) for biomedical research.

## SUPPLEMENTARY MATERIALS TABLES


