# Supplementary material for: Skeletal muscle depletion predicts survival of patients with advanced biliary tract cancer undergoing palliative chemotherapy
Source: Oncotarget. 2017 Jun 2;8(45):79441–52. doi: 10.18632/oncotarget.18345 (PMC5668056; doi:10.18632/oncotarget.18345)
Supplement: Supplementary file 1 [file oncotarget-08-79441-s001.pdf]

Skeletal muscle depletion predicts survival of patients with advanced biliary tract cancer undergoing palliative chemotherapy

Supplementary Materials

Supplementary Table 1: Chemotherapy regimen

	N (%)	Median Duration (Months)	Median Cycles	Regimen
Gemcitabine + Platinum	209 (39.9 %)	4.70 months	4.1	
(Gemcitabine + Cisplatin)	199 (95.2 %)	4.73 months	4.1	Gemcitabine: 1,200 mg/m2 [D1,8], Cisplatin: 60 mg/m2 q3 weeks (before 2012.7) Gemcitabine: 1,000 mg/m2 [D1,8], Cisplatin: 25 mg/m2 [D1,8] q3 weeks (after 2012.8)
(Gemcitabine + Oxaplatin)	10 (4.8 %)	3.60 months	5.5	Gemcitabine: 1,000 mg/m2, Oxaliplatin:100 mg/m2 q2 weeks
5-FU + Platinum	198 (37.8 %)	4.43 months	4.9	
(TS-1 + Cisplatin)	139 (70.2 %)	4.38 months	4.7	TS-1: 40 mg/m2 po bid [D1-14], Cisplatin: 60 mg/m2 q3 weeks
(5-FU + Cisplatin)	43 (21.7 %)	6.52 months	3.8	5-FU: 1,200 mg/m2 [D1-4], Cisplatin:60 mg/m2 D1 q3 weeks
(Xeloda + Oxaplatin)	8 (4.5 %)	3.90 months	4.9	Xeloda: 1000 mg/m2 po bid[D1-14], Oxaliplatin: 130 mg/m2 q3 weeks
(TS-1 + Oxaplatin)	6 (3 %)	2.43 months	2.2	TS-1: 40 mg/m2 po bid [D1-14], Oxaliplatin: 130 mg/m2 q3 weeks
(Xeloda + Cisplatin)	1 (0.5 %)	3.80 months	5.0	Xeloda: 1250 mg/m2 po bid [D1-14], Cisplatin: 130 mg/m2 q3 weeks
Others	117 (22.3 %)	3.53 months	1.7	
(iFAM)	28 (23.9 %)	2.50 months	1.9	5-FU: 800 mg/m2 [D1-5], ADR:30 mg/m2 [D1], MMC: 8 mg/m2 [D1] q4 weeks
(5-FU based CCRTx)	14 (12.0 %)	2.8 months	1.5	5-FU: 375 mg/m2 [D1-3] & [D29-31] during RTx
(TS-1)	14 (12.0 %)	5.2 months	1.2	TS-1: 40 mg/m2 po bid [D1-28] q6 weeks
(FOLFIRI)	2 (1.7 %)	2.2 months	2.5	Irinotecan; 180 mg/m2 [D1], 5-FU:400 mg/m2 iv push [D1, 2]
(Xeloda)	21 (18.1 %)	5.7 months	1.4	Xeloda: 1250 mg/m2 bid [D1-14] q3 weeks
(FOLFOX)	1 (0.9%)	Not Acessable (F/U loss)	Not Acessable (F/U loss)	Oxaplatin: 85 mg/m2 [D1], 5-FU: 400 mg/m2 [D1] iv push, 5-FU: 2400 mg/m2 [D1] for 46 hrs [D1] q2weeks
(Gemcitabine)	23 (19.7%)	2.7 months	2.0	Gemcitabine: 1000 mg/m2 [D1,8,15] q4 weeks
(Gemcitabine + 5-FU)	7 (6.0%)	1.4 months	2.0	Gemcitabine: 1200 mg/m2 [D1,8], 5-FU:1,200 mg/m2 [D1-4] q3 weeks
(Paclitaxel + Cisplatin)	1 (0.9%)	4.2 months	6.0	Paclitaxel: 175 mg/m2 [D1], Cisplatin:60 mg/m2 [D1] q3 weeks
(Xeloda + Gemcitabine)	2 (1.8%)	6.8 months	1.5	Gemcitabine; 1,000 mg/m2 [D1,8], Xeloda:750 mg/m2 po bid [D1-14] q3 weeks
(TS-1 + Gemcitabine)	2 (1.8%)	8.1 months	11.5	Gemcitabine; 1,000 mg/m2 [D1,8], TS-1:50 mg bid q3 weeks

Supplementary Table 2: Prevalence of sarcopenia for each body mass index subgroup

Characteristic		BMI < 20 kg/m² (*N* = 82)		BMI 20–24.9 kg/m² (*N* = 312)		BMI ≥ 25 kg/m² (*N* = 116)		Total (*N* = 510)	p^a
		Low skeletal muscle mass	Non-low skeletal muscle mass	Low skeletal muscle mass	Non-low skeletal muscle mass	Low skeletal muscle mass	Non- low skeletal muscle mass		
Age	< 60	20 (74.1%)	7 (25.9%)	43 (36.8%)	74 (63.2%)	3 (9.4%)	29 (90.6%)	176	0.002
	≥ 60	35 (85.4%)	6 (14.6%)	92(59.4%)	63 (40.6%)	13 (20.0%)	52 (80.0%)	261	
Gender	Male	44 (81.5%)	10 (18.5%)	94 (54.0%)	80 (46.0%)	10 (17.7%)	50 (83.3%)	288	0.071
	Female	11 (78.6%)	3 (21.4%)	41 (41.8%)	57 (58.2%)	6 (16.2%)	31 (83.8%)	149	
Total		55(80.9%)	13 (19.1%)	135 (49.6%)	137 (50.4%)	16(16.5%)	81 (83.5%)		

BMI, body mass index.
[a]*p* values were calculated using the Fisher exact test.

Supplementary Table 3: Risk factors for decrease in skeletal muscle index

		HR	95% CI	p^a
Sex	Male	1.598	0.826–3.089	0.164
Age (years)	> 60	0.834	0.452–1.541	0.562
Primary tumor site				0.008
	ICC	4.964	1.297–18.990	0.019
	GB Ca	7.704	1.981–29.958	0.003
	Extrahepatic BTC	10.958	2.540–47.273	0.001
	AoV Ca	1		
ECOG PS	> 2	3.572	1.068–11.945	0.039
Initial SMI†	Low skeletal muscle mass	0.611	0.309–1.210	0.158
Initial BMI				
	< 20	1		0.639
	20 < BMI < 24.99	0.731	0.284–1.880	0.516
	BMI > 25	0.978	0.313–3.050	0.969
Change in BMI‡	Decreased	2.650	1.149–6.109	0.022
Chemotherapy response	Progression	1.614	0.867–3.002	0.131
Disease status	Metastasis	2.013	0.999–4.058	0.05

ICC,intrahepatic cholangiocarcinoma;GB Ca,gallbladder cancer; extrahepatic BTC, extrahepatic biliary tract cancer; AoV Ca,ampulla of Vater cancer; ECOG PS, Eastern Cooperative Oncology Group performance status; BMI, body mass index; HR, hazard ratio; CI, confidential interval.

† Low skeletal muscle mass: males < 48.5 cm^2/m^2, females < 39.5 cm^2/m^2.

‡ Change in BMI: decreased < −7 kg/m^2.

ap values were calculated using the Logistic regression.

Supplementary Table 4: Changes in body composition during first line chemotherapy by primary tumor site

	ICC *N* (%)	GB Ca *N* (%)	Extrahepatic BTC *N* (%)	AVO Ca *N* (%)	*p* value
Change in BMI Mean	−1.514	−2.224	−1.542	−3.349	0.50[b]
Kg/m^2 (%) SD	6.231	6.211	7.003	8.171	
Change in BMI					0.628[a]
Decreased	18 (14.1%)	15 (16.1%)	9 (20.9%)	8 (21.1%)	
Maintained	110 (85.9%)	78 (83.9%)	34 (79.1%)	30 (78.9%)	
Change in SMI Mean	−5.303	−6.040	−7.003	−1.17	0.085[b]
Cm^2/m^2 (%) SD	10.826	9.845	11.369	8.108	
Change in SMI					0.015[a]
Decreased	41 (33.1%)	39 (39.8%)	20 (45.5%)	4 (12.5%)	
Maintained	83 (66.9%)	59 (60.2%)	24 (54.5%)	28 (87.5%)	

†Change in BMI: maintained ≥ −7% kg/m^2, decreased < −7% kg/m^2

‡Change in SMI: maintained ≥ −7 % cm^2/m^2; decreased < −7 %cm^2/m^2.

[a]*p* values were calculated using the Fisher exact test. [b]*p* values were calculated using the krukal-Walis test.

Supplementary Table 5: Factors associated with OS (Subgroup Analysis according to ICC)

		Univariate analysis			Mutivariate		
		**OS , months**	**95% CI**	***p* value**	**HR**	**95% CI**	***p* value**
ICC ($N = 231$)							
Low skeletal muscle mass	Yes	7.00	5.804–8.196	< 0.001	1.659	1.011–2.723	0.045
	No	11.00	9.190–12.810		1		
BMI at diagnosis				0.736			0.550
	< 20 kg/m^2	7.00	5.419–8.581		1		
	20–24.9 kg/m^2	9.00	7.614–10.386		0.715	0.377–1.356	0.305
	> 25 kg/m^2	9.00	6.858–11.142		0.8 77	0.379–2.029	0.759
Change in BMI	Decreased	8.00	5.921–10.079	0.205	1.394	0.696–2.791	0.349
	Maintained	10.00	8294–11.706		1		
Change in SMI	Decreased	6.00	5.037–6.963	0.002	1.898	1.157–3.115	0.011
	Maintained	12.00	10.720–13.280		1		

[a]*p* values were calculated using the Cox-proportional hazard model, adjusted with age, 1st chemotherapy, PS, disease conrolled rate.

Supplementary Table 6: Factors associated with OS (completion rate)

		Univariate analysis			Mutivariate		
		OS, months	95% CI	*p* value	HR	95% CI	*p* value
Gender	Male	9.67	7.864–11.269	0.440	1		0.833
	Female	9.10	7.783–10.417		0.963	0.681–1.362	
Age	< 60	9.40	8.182–10.618	0.128	1		0.152
	> 60	9.50	7.712–11.288		1.250	0.921–1.696	
Primary tumor site				0.257			0.118
	ICC	9.40	7.332–11.468		1		0.118
	GB Ca	8.90	6.915–10.885		0.978	0.687–1.392	0.901
	Extrahepatic BTC	8.83	6.920–10.747		0.697	0.445–1.090	0.113
	AoV Ca	14.70	7.761–21.639		0.584	0.341–1.000	0.050
ECOG PS	0–1	9.50	8.082–10.918	0.015	1		0.299
	> 2	5.80	1.400–10.200		1.373	0.755–2.496	
Low skeletal muscle mass	Yes	7.80	6.589–9.011	0.019	1.614	1.153–2.258	0.005
	No	10.70	9.226–12.174		1		
Disease status	Locally advanced	8.67	7.654–9.679	0.928	1		0.993
	Metastatic	9.90	8.138–11.662		0.998	0.713–1.399	
Chemotherapy	Gemcitabine + Platinum	8.60	7.39–9.81	0.805	1		0.829
	5FU + Platimun	10.23	8.63–11.94		1.088	0.787–1.504	0.609
	Others	10.70	7.69–13.71		1.123	0.716–1.761	0.612
BMI at diagnosis				0.051			0.023
	< 20 kg/m2	6.83	5.670–7.996		1		
	20–24.9 kg/m2	10.33	8.730–11.937		0.695	0.448–1.078	0.105
	> 25 kg/m2	8.63	7.545–9.722		1.163	0.676–2.002	0.586
Change in BMI	Decreased	8.77	6.951–1.583	0.061	1		0.153
	Maintained	9.97	8.362–11.571		0.736	0.483–1.121	
Change in SMI	Decreased	6.60	5.845–7.355	< 0.001	1		< 0.001
	Maintained	12.07	10.911–13.222		0.372	0.268–0.516	
Best response	Controlled	12.17	10.960–13.373	< 0.001	1		< 0.001
	Progression	8.80	5.881–7.319		2.695	1.947–3.731	

ICC,intrahepatic cholangiocarcinoma;GB Ca,gallbladder cancer;extrahepatic BTC, extrahepatic biliary tract cancer;AoV Ca,ampulla of Vater cancer; ECOG PS, Eastern Cooperative Oncology Group performance status; BMI, body mass index; SMI, skeletal muscle index; OS, overall survival; HR, hazard ratio; CI, confidential interval. [†] controlled: complete response, partial response, and stable disease.

Supplementary Table 7: Correlation of BMI and SMI at diagnosis

		BMI at diagnosis			
		**< 25 kg/m² (*N* = 175)**	**≥ 25 kg/m² (*N* = 50)**		
SMI at diagnosis	Low skeletal muscle mass, *N* = 104	HR:1.885 (95% CI:1.283–2.768)<br>*p* = 0.001[a], OS = 7.00 months, *N* = 90	HR:1.748 (95% CI:0.777–3.929)<br>*p* = 0.177[a], OS = 6.00 months, *N* = 9	*p* = 0.330 [a,c]	*p* = 0.011[a]
	Non- low skeletal muscle mass, *N* = 97	HR:1 reference<br>OS = 11.00 months, N=62	HR:1.279 (95% CI:0.767–2.132)<br>*p* = 0.345[a], OS = 10.00 months, *N* = 34	*p* = 0.359 [a,d]	
SMI males, (*N* = 178)	Low skeletal muscle mass, *N* = 79	HR:2.085 (95% CI:1.315–3.307)<br>*p* = 0.002[b], OS = 7.00 months, *N* = 71	HR: 2.561 (95% CI:0.875–7.494)<br>*p* = 0.086[b], OS = 6.00 months, *N* = 6	*p* = 0.703 [b,c]	*p* = 0.016[b]
	Non- low skeletal muscle mass, *N* = 72	HR:1 Reference<br>OS= 12.00 months, *N* = 45	HR:1.754 (95% CI:0.951–3.234)<br>*p* = 0.072[b], OS = 10.00 months, *N* = 26	*p* = 0.099 [b,d]	
SMI female, (*N* = 53)	Low skeletal muscle mass, *N* = 25	HR: 1.785 (95% CI:0.796–4.001)<br>*p* = 0.159 [b], OS = 6.00 months, *N* = 19	HR:1.510 (95% CI:0.411–5.548)<br>*p* = 0.535 [b], OS = 7.00 months, *N* = 3	*p* = 0.542 [b,c]	*p* = 0.486 [b]
	Non- low skeletal muscle mass, *N* = 25	HR: 1 Reference<br>OS = 9.00 months, *N* = 17	HR: 0.956 (95% CI:0.311–2.941)<br>*p* = 0.937[b],OS = 10.00 months, *N* = 8	*p* = 0.902 [b,d]	

[a]*p* values were calculated using the Cox-proportional hazard model, age,gender and PS.
[b]*p* values were calculated using the Cox-proportional hazard model, adjusted with age, PS
[c]*p* values were calculated within low skeletal muscle mass group. [d]*p* values were calculated within non- low skeletal muscle mass group.
(Subgroup Analysis according to ICC).

Supplementary Table 8: Factors associated with OS (previous cut off value)

		Univariate analysis			Mutivariate		
		OS, months	95% CI	*p* value	HR	95% CI	*p* value
Gender	Male	9.00	8.110–9.890	0.837	1		0.249
	Female	9.00	8.039–9.961		1.252	0.855–1.834	
Age	< 60	10.00	9.123–10.877	0.033	1		0.421
	> 60	8.00	7.142–8.858		1.130	0.839–1.523	
Primary tumor site				0.106			0.108
	ICC	9.00	7.993–10.007		1		
	GB Ca	9.00	7.924–10.076		0.900	0.632–1.282	0.560
	Extrahepatic BTC	9.00	7.829–10.171		0.744	0.477–1.159	0.191
	AoV Ca	12.00	8.863–15.137		0.539	0.321–0.907	0.020
ECOG PS	0–1	10.00	9.186–10.814	< 0.001	1		0.479
	> 2	5.00	3.647–6.353		1.238	0.686–2.237	
Low skeletal muscle mass	Yes	8.00	7.072–8.928	0.004	1.588	1.082–2.331	0.018
	No	10.00	8.901–11.099		1		
Disease status	Locally advanced	9.00	8.029–9.971	0.509	1		0.182
	Metastatic	9.00	8.115–9.885		1.258	0.898–1.763	
Chemotherapy	Gemcitabine+ Platinum	9.00	7.920–10.080	0.154	1		0.559
	5FU+Platimun	9.00	7.821–10.179		0.936	0.685–1.280	0.680
	Others	9.00	7.644–10.356		1.236	0.758–2.016	0.396
BMI at diagnosis				0.345			0.183
	< 20 kg/m2	8.00	6.338–9.662		1		
	20–24.9 kg/m2	9.00	8.067–9.933		0.861	0.553–1.340	0.506
	> 25 kg/m2	9.00	7.879–10.121		1.268	0.730–2.203	0.399
Change in BMI	Decreased	8.00	6.594–9.406	0.003	1		0.188
	Maintained	10.00	8.909–11.091		0.760	0.505–1.144	
Change in SMI	Decreased	7.00	6.291–7.709	< 0.001	1		< 0.001
	Maintained	12.00	10.776–13.224		0.468	0.340–0.643	
Best response	Controlled	11.00	10.007–11.993	< 0.001	1		0.007
	Progression	6.00	5.335–6.665		1.691	1.158–2.469	

ICC,intrahepatic cholangiocarcinoma;GB Ca,gallbladder cancer;extrahepatic BTC, extrahepatic biliary tract cancer;AoV Ca,ampulla of Vater cancer; ECOG PS, Eastern Cooperative Oncology Group performance status; BMI, body mass index; SMI, skeletal muscle index; OS, overall survival; HR, hazard ratio; CI, confidential interval. † controlled: complete response, partial response, and stable disease.

Supplementary Table 9: Factors associated with OS (Validation)

Characteristics	Patients (N = 62)
Age — yr	
Median	62
Range	40–78
Sex-no (%)	
Male	37 (59.7%)
Female	25 (40.3%)
Primary tumor site — no. (%)	
ICC	16 (25.9%)
GB Ca	22 (35.5%)
Extrahepatic BTC	22 (35.5%)
AoV Ca	2 (3.2%)
ECOG PS— no. (%)	
0	14 (22.6%)
1	48 (77.4%)
CEA (ng/ml) median (range)	2.9 (0.5–104.2)
CA 19-9 (U/ml) median (range)	121.5 (2.0–36000)
Total bilirubin (mg/dl) median (range)	0.7 (0.3–4.3)
Albumin (mg/dl) median (range)	3.90 (2.7–4.6)
1st chemotherapy	
Gemcitabine+Cisplatin (Gemcitabine; 1,000 mg/m2 [D1,8], Cisplatin; 25 mg/m2 [D1, 8] q3 weeks)	61 (98.4%)
Xelox (Oxaplatin; 130 mg/m2 [D1], Xeloda;1000 mg/m2 bid [D1-14] q2 weeks)	1 (0.6%)
Overall survival (months)	p = 0.664
Low skeletal muscle mass	11.93 months
Non-low skeletal muscle mass	13.97 months

ICC,intrahepatic cholangiocarcinoma;GB Ca,gallbladder cancer;extrahepatic BTC, extrahepatic biliary tract cancer;AoV Ca,ampulla of Vater cancer; ECOG PS, Eastern Cooperative Oncology Group performance status
